# Giant thymoma successfully resected via anterolateral thoracotomy: a case report

**DOI:** 10.1186/s13019-015-0321-y

**Published:** 2015-09-01

**Authors:** Takahiro Saito, Takashi Makino, Yoshinobu Hata, Satoshi Koezuka, Hajime Otsuka, Kazutoshi Isobe, Naobumi Tochigi, Kazutoshi Shibuya, Sakae Homma, Akira Iyoda

**Affiliations:** 1 Division of Chest Surgery, Toho University School of Medicine, Tokyo, Japan; 2 Division of Respiratory Medicine, Toho University School of Medicine, Tokyo, Japan; 3 Department of Surgical Pathology, Toho University School of Medicine, Tokyo, Japan

**Keywords:** Surgery, Giant thymoma, Thymoma, Anterolateral incision

## Abstract

The appropriate surgical approach for a large mediastinal tumor is controversial. Median sternotomy is the standard approach for thymomas. We herein report the case of a giant thymoma, 13 cm in diameter, surgically resected via anterolateral incision. Subsequent thymectomy was performed via thoracoscopy. The resected specimen was a WHO type AB thymoma, Masaoka stage I, without capsular invasion. The anterolateral incision was less invasive and more versatile in the present case, as the incision could be extended to a hemiclamshell or posterolateral incision depending on exposure and relationship to adjacent organs and vascular structures.

## Background

Thymomas are rare neoplasms with an indolent growth pattern and present with various clinical symptoms [1]. They are commonly found in the anterior mediastinum. Complete surgical resection is the mainstay of treatment. While median sternotomy has been the standard approach for thymectomy, the best incision is controversial for so-called giant thymomas [2]. Here we report the case of a giant thymoma in the anterior-inferior mediastinum successfully resected with additional thymectomy via anterolateral thoracotomy.

## Case presentation

**Fig. 1:**
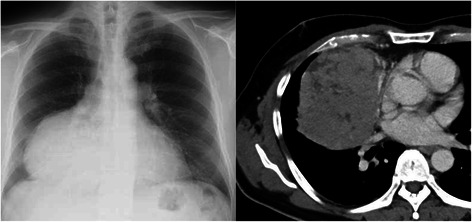
Chest x-ray shows a giant mass in the right lower lung field (*left*). Chest CT shows a mass measuring 13 x 10 cm in diameter, in contact with the right inferior pulmonary vein (*right*)

A 45-year-old man was referred to our hospital due to a routine chest x-ray showing an abnormal shadow in the right lower lung field (Fig. 1). He had no obvious symptoms, except for slight dyspnea on exertion for three months. He had no smoking history and no significant medical history. No previous chest x-rays were available. Chest CT-scan showed a well-defined mass 13 x 10 cm in diameter, in contact with the diaphragm, pericardium, right inferior pulmonary vein, and superior vena cava (Fig. 1). The tumor showed heterogenous contrast effect. F18-fluorodeoxyglucose positron emission tomography (FDG-PET) showed abnormal FDG uptake with maximum standardized uptake value of 4.2. Laboratory examination showed normal serum levels of alpha fetoprotein (2.4 ng/ml), human chorionic gonadotropin beta (<0.2 ng/ml) and anti-acetylcholine receptor antibody. Differential diagnosis included thymoma, thymic carcinoma, and a germ cell tumor; surgical resection was thus recommended. Preoperative needle biopsy was not performed because of the risk of dissemination or bleeding. As the tumor showed possible invasion into the superior vena cava, inferior pulmonary vein and diaphragm, we elected to perform an anterolateral thoracotomy in the fifth intercostal space in the semi-lateral decubitus position, which could be extended to a posterolateral thoracotomy or hemi-clamshell thoracotomy depending on the relationship of the tumor to the inferior pulmonary vein or superior vena cava, respectively.

Thoracoscopic examination through the eighth intercostal space revealed no adhesions or pleural disease. An anterolateral incision, 20 cm in length, was made in the fifth intercostal space. The tumor was excised from the anterior mediastinal fat tissue and thymus. Dense adhesions of the tumor to the pericardium were sharply peeled off, and the tumor was resected without involvement of the superior vena cava or inferior pulmonary vein. Intraoperative frozen section diagnosed the tumor as a thymoma; thymectomy was thus performed through the same incision via thoracoscopy. The adherent portion of the pericardium was excised and reconstructed with the use of a Gore-Tex pericardial patch.

**Fig. 2:**
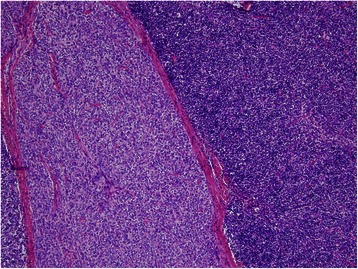
Microscopic examination revealed a WHO type AB thymoma, without capsular invasion

The resected specimen was 13 × 11.8 × 8 cm, showing a well encapsulated tumor with a lobulated appearance separated by fibrous bands. Microscopic examination revealed the tumor to be composed of a lymphocyte-associated area and a spindle cell-dominant area (Fig. 2), which was diagnosed as World Health Organization (WHO) Type AB thymoma without capsular invasion (Masaoka stage I). The postoperative course was uneventful and the patient is free of recurrence 12 months after the surgery.

### Discussion

The optimal surgical approach for giant thymomas is a point of controversy (Table 1). Two cases of giant thymoma resected via anterolateral thoracotomy have been reported [2, 3]. One case was an ectopic pleural thymoma measuring 20 cm in size located in the lower portion of the right thoracic cavity, with adhesions to the pulmonary pleura of the right lower lobe and central part of the diaphragm [3]. The other case was an anterio-inferior mass weighing 1705 g, which was attached to the mediastinal pleura via a small vascular bundle. This was easily removed after transection of the pedicle, and additional thymectomy was performed [2]. In the present case, we preferred the anterolateral approach because the incision could be extended to either a posterolateral approach in the case of adhesions to the inferior pulmonary vein, or a hemiclamshell approach in case of adhesions to the superior vena cava. Additional thymectomy was successfully performed via the same incision via thoracoscopy, thus avoiding the need for a second operation.

**Table 1:** Surgical approach for giant thymoma

Approach	*n*	Cases	Remarks
Median sternotomy [reference 4–6]	3	Anterior masses	Suitable for invasion into innominate vein
			Possible blind spot caused by anterior mass
Hemiclamshell [reference 12–14]	3	Large masses occupying more than half of thorax	Easy access to the mediastinum and hilum
			Relatively invasive
Posterolateral [reference 8, 9]	2	Masses close to the diaphragm	Suitable for inferior mediastinal masses
			Requires thymectomy at second operation
			Unsuitable for antero-superior mediastinal masses
Anterolateral [reference 2, 3]	2	Antero-inferior masses	Possible to extend the incision posteriorly or with median sternotomy
		Ectopic mass	Unsuitable in cases that are unstable in the decubitus position
Clamshell [reference 7]	1	Masses with bleeding	Quick access to the hilum and tumor control
			Invasive

While median sternotomy is the standard approach for thymomas, only three cases of giant thymomas resected via median sternotomy have been reported [4–6]. Median sternotomy was suitable for one case with invasion into the innominate vein [4], but access to the hilum [7] or posterior thorax can be difficult in cases of giant thymomas. A hemiclamshell approach is a reasonable approach for giant thymomas [2], but is relatively more invasive compared with the other approaches. The clamshell incision, widely used in lung transplantation procedures, was selected in an emergency operation for a patient in shock secondary to spontaneous rupture of a giant thymoma, thought to be a giant sarcoma in close contact with the pulmonary artery [7]. The clamshell approach enables rapid tumor control and easy access to the hilum. A posterolateral approach was reported in two cases [8, 9]. One case was an ectopic pleural thymoma, preoperatively suspected to be a solitary fibrous tumor, and a subsequent transcervical thymectomy was not performed. In the other case, the remaining thymus gland portions were removed through a median sternotomy at a second operation [9]. Anterolateral thoracotomy is less invasive than the clamshell and hemiclamshell approaches, and may be appropriate for cases of giant thymoma.

While the size of the thymoma has been reported to be a significant prognostic factor from experienced single centers [10, 11], sporadic case reports of giant thymomas larger than 13 cm consisted of 4 cases of WHO type A, 6 cases of type AB (including the present case), and 2 cases of type B1 [2–9, 12–14]. Although the five year survival for thymomas more than 10 cm is reported to be 72 % [10], successfully resected giant thymomas tend to be low-grade [12].

## Conclusion

In the present case, surgical resection for a giant thymoma and additional thymectomy were successfully performed via an anterolateral approach, which is relatively less invasive and more versatile due to the ability to extend the incision posteriorly or to add a median sternotomy.

## Consent

Written informed consent was obtained from the patient for publication of this case report and any accompanying images. A copy of the written consent is available for review by the Editor-in-Chief of this journal.

## Abbreviations

FDG-PET: F18-fluorodeoxyglucose positron emission tomography
WHO: World Health Organization
